# A comparison study on the clinical effects of foscarnet sodium injection and interferon on human immunodeficiency virus-infected patients complicated with herpes zoster

**DOI:** 10.12669/pjms.312.6536

**Published:** 2015

**Authors:** Yuan Yuan-Qu, Ming Xiong-Pu, Jing Xiao-Kang, Xia Cai-An

**Affiliations:** 1 Yuan-Yuan Qu, Department of Dermatology and Venereology, Anhui Medical University, Hefei, Anhui Province, China; 2 Xiong-Ming Pu, Department of Dermatology, People’s Hospital of Xinjiang, Urumqi, Xinjiang Uygur Autonomous Region, China; 3 Xiao-Jing Kang, Department of Dermatology, People’s Hospital of Xinjiang, Urumqi, Xinjiang Uygur Autonomous Region, China; 4 Cai-Xia An, Department of Dermatology, People’s Hospital of Xinjiang, Urumqi, Xinjiang Uygur Autonomous Region, China

**Keywords:** HIV infection, Herpes zoster, Foscarnet sodium, Interferon

## Abstract

**Objective:**

To compare the clinical effects of foscarnet sodium injection and interferon on human immunodeficiency virus (HIV)-infected patients complicated with herpes zoster.

**Methods:**

Ninety HIV-infected patients complicated with herpes zoster were divided into a treatment group and a control group that were both treated routinely first. Then the control group and treatment group were administered with interferon and foscarnet sodium injection respectively for four consecutive weeks.

**Results:**

After four weeks, the effective rates of the treatment and control groups were 95.6% and 80.0% respectively, which were significantly different (P < 0.05). The pain scores of the two groups were similar before treatment, but the scores of the treatment group were significantly lower than those of the control group two and four weeks after treatment (P < 0.05) as well as were significantly lower than those before treatment (P < 0.05). The numbers of CD4+ cells and the contents of IL-2 of both groups two and four weeks after treatment significantly exceeded those before treatment (P < 0.05), with significant inter-group differences also (P < 0.05). Two and four weeks after treatment, the treatment group scored significantly higher in physical activity, energy, sleep, social life and emotional reaction than the control group did (P < 0.05).

**Conclusions:**

HIV-infected patients are prone to being complicated with herpes zoster. Compared with interferon, foscarnet sodium injection better improves the clinical outcomes by effectively relieving pain and by regulating immune mediated inflammatory diseases, thus boosting the prognostic quality of life.

## INTRODUCTION

Currently, human immunodeficiency virus (HIV) and acquired immunodeficiency syndrome (AIDS) have been severely endangering the whole world as public health issues.^1^,^2^ Epidemiological studies have shown that of about seven hundred thousand HIV-infected patients, approximately one hundred thousand ones suffered from AIDS that was mainly transmitted sexually or by intravenous drug injection.^3^-^5^ Herpes zoster, as an acute infectious skin disease induced by Varicella-Zoster virus (VZV), is typified by distribution of rash along peripheral nerve segments and neuralgia. During early infection, the virus lurks in the dorsal root neurons persistently, which, upon cellular immune dysfunction, is activated to induce ganglion inflammation, necrosis and acute skin damages.^6^

The patients with herpes zoster undergo long-term pain, depression and decrease in quality of life. There remain no eligible drugs for treating HIV plus herpes zoster, which are thus now routinely treated by anti-inflammation, infection prevention, disease course shortening and symptom-targeting strategies with antiviral agents, interferon, corticoids, transfer factors and B vitamins, etc.^7^ Interferon can inhibit virus from damaging nerves, control acute-phase symptoms and prevent postherpetic neuralgia, but it may lead to adverse reactions and high recurrence rates.^8^

With wide application of highly active anti-retroviral therapy in AIDS patients, anti-HIV treatment has obtained triumphs, and the transmission of HIV has been well controlled. However, resistant viruses have increased due to long-term individual use of a certain drug.^9^ Foscarnet sodium injection is a well-known, broad-spectrum antiviral agent. By directly binding to the pyrophosphate binding sites of RNA and DNA polymerases, foscarnet, as a non-nucleoside analog of pyrophosphate, non-competitively inhibits enzyme activity with nucleotide substrates.^10^ Besides, it selectively suppresses virus-specific DNA polymerase and well inhibits hepatitis B virus replication.^11^

In this study, we compared the clinical effects of foscarnet sodium injection and interferon on HIV-infected patients complicated with herpes zoster.

## METHODS

### Subjects

Ninety HIV-infected patients complicated with herpes zoster who were treated in our hospital from February 2008 to December 2013 were selected in this study.

### Inclusion Criteria

In accordance with “Diagnostic criteria and principles of management of asymptomatic HIV/AIDS” (P. R. China, 2001 edition); expected survival rime > 1 year; with herpes and severe neuropathic pain within 1-7 days, without receiving antiviral or analgesic therapy; with written consent form to accept all possible therapies; 18-65 years old.

### Exclusion standards

Severe liver and renal insufficiencies, or those complicated with severe primary cardiovascular, lung and hematopoietic system diseases; psychiatric patients; pregnant or lactating women; allergic to the drugs used herein; with primary immunodeficiency, hormone- or chemotherapy-induced secondary immunodeficiencies, or hematological diseases. Platelet count < 60×10^9^/L. The patients were divided into a treatment group and a control group by random draw (n=45), and their gender, age, disease course, education time, body mass index and history of drug injection were similar (P > 0.05) (Table-I).

**Table-I:** Baseline clinical data.

Group	Gender (male/female)	History of drug injection (case)	Age (years old)	Disease course (year)	Education time (year)	Body mass index (kg/m^2^)
Treatment group (n=45)	25/20	21(46.7%)	46.44±2.87	4.22±0.45	12.73±6.13	19.78±5.22
Control group (n=45)	24/21	20(44.4%)	46.39±3.13	4.23±0.65	12.77±5.33	19.36±5.33
χ^2^	0.078	0.50				
t			0.065	0.009	0.067	0.302
P	>0.05	>0.05	>0.05	>0.05	>0.05	>0.05

### Treatment methods

### Basic treatment

1.2 g reduced glutathione; 150 mg diammonium glycyrrhizinate, qd, intravenous infusion; oral administration of valacyclovir hydrochloride, 300 mg per time, bid; oral administration of vitamin B1, 10 mg, tid.

### Control group

Based on the basic treatment, 50 million U IFN α-2b interferon (Jiangsu Chia Tai Tianqing Pharmaceutical Co., Ltd.) was intramuscular injected, qd.

### Treatment group

Based on the basic treatment, 3.0 g foscarnet sodium injection (Jiangsu Chia Tai Tianqing Pharmaceutical Co., Ltd.) was intravenously infused, bid. Both groups were treated for four consecutive weeks, with seven days as a course of treatment. Serum and proteins were used to support treatment, but immunomodulators, jaundice-removing agents, other antiviral drugs or plasmapheresis were not employed.

### Observation indices

Determination of therapeutic effects: Evaluation was performed four weeks after treatment. Markedly effective: Disappearance of rash and clinical signs (Rash means skin changes such as color, appearance, or texture; clinical signs mean pain, depression and decrease in quality of life), and significantly mitigated pain and itching; effective: disappearance of 30% rash, significantly decreased clinical signs, and mitigated pain and itching; ineffective: unable to reach the above standards.

### Pain rating

Degree of pain was rated before as well as two and four weeks after treatment by visual analogous scale, with 0 being painless and 100 being the maximum pain that patients could imagine. The scale was completed by patients.

### Investigation on quality of life

Nottingham health profile, which includes physical activity, energy, sleep, social life and emotional reaction, was used to investigate the patients two and four weeks after treatment. From 0 to 100, a higher score means better quality of life. The profile was highly reliable and valid.

### Comparisons of inflammatory and immune cytokines

Before as well as two and four weeks after treatment, 5 mL of venous blood was collected and stored in a -20°C refrigerator after separating serum at low temperature. Interleukin-2 (IL-2) content was detected by polyclonal antibody-based sandwich ELISA according to the instructions of kit (Genzyme, USA). CD4+ cells were counted according to instructions by experienced personnel in our hospital.

### Statistical analysis

All data were analyzed by SPSS 17.0. The categorical data were expressed as (x ± s), and intra-group and inter-group comparisons were performed by using t test and independent analysis of variance. The numerical data were compared by Chi-square analysis. P < 0.05 was considered statistically significant.

## RESULTS

### Clinical effects

Four weeks after treatment, the effective rate of the treatment group (95.6%) was significantly higher than that of the control group (80.0%) (P < 0.05) (Table-II).

**Table-II:** Clinical effects (n).

Group	Case No.	Markedly effective	Effective	Ineffective	Overall effective rate
Treatment group	45	35	8	2	95.6%
Control group	45	15	21	9	80.0%
χ²					7.193
P					<0.05

### Pain scores

The pain scores of the two groups were similar before treatment, but the scores of the treatment group were significantly lower than those of the control group two and four weeks after treatment (P < 0.05) as well as were significantly lower than those before treatment (P < 0.05) (Table-III).

**Table-III:** Pain scores at different time points before and after treatment (point, x ± s).

Group	Case No. (n)	Before	Two weeks after	Four weeks after
Treatment group	45	80.44±7.13	12.87±5.33	6.89±3.12
Control group	45	80.65±6.09	34.17±5.11	20.11±4.01
t		0.028	22.863	14.992
P		>0.05	<0.05	<0.05

### Changes of inflammatory and immune cytokines

The numbers of CD4+ cells and the contents of IL-2 of both groups two and four weeks after treatment significantly exceeded those before treatment (P < 0.05), with significant inter-group differences also (P < 0.05) (Table-IV).

**Table-IV:** Numbers of CD4+ cells and IL-2 contents at different time points before and after treatment (x ± s).

Group	Case No. (n)	Number of CD4+ cells (count/μl)			IL-2 content (ng/L)		
		Before	Two weeks after	Four weeks after	Before	Two weeks after	Four weeks after
Treatment group	45	109.37±45.33	302.76±60.27	338.65±59.09	445.22±90.37	879.68±78.03	1165.98±109.33
Control group	45	111.76±46.01	198.26±45.11	230.68±63.45	448.26±43.09	600.38±61.34	876.63±78.26
t		0.284	17.367	9.367	0.391	34.762	21.093
P		>0.05	<0.05	<0.05	>0.05	<0.05	<0.05

### Evaluation on quality of life

Two and four weeks after treatment, the treatment group scored significantly higher in physical activity, energy, sleep, social life and emotional reaction than the control group did (P < 0.05) (Table-V).

**Table-V:** Scores in quality of life at different time points before and after treatment (point, x ± s).

Group	Case No. (n)	Two weeks after					Four weeks after				
		Physical activity	Energy	Sleep	Social life	Emotional reaction	Physical activity	Energy	Sleep	Social life	Emotional reaction
Treatment group	45	78.92±7.49	69.37±8.09	72.63±6.01	76.98±5.11	77.18±8.31	91.36±6.13	92.56±7.20	90.78±6.22	95.22±7.02	93.56±6.11
Control group	45	67.99±5.09	56.62±6.44	61.68±7.77	65.26±6.81	59.33±7.22	80.36±5.19	78.68±4.89	82.68±7.29	80.63±6.66	82.56±7.23
t		11.377	13.847	11.003	11.084	18.467	11.044	14.665	8.462	15.882	11.662
P		<0.05	<0.05	<0.05	<0.05	<0.05	<0.05	<0.05	<0.05	<0.05	<0.05

## DISCUSSION

AIDS is a chronic infectious disease caused by HIV that belongs to the genus *Lentivirus* of the family *Retroviridae* and exists in most body fluids and tissues of HIV-infected and AIDS patients. This virus is mainly transmitted by sexual contact and blood in addition to mother-to-child transmission. Upon HIV invasion, the immune system of human body is attacked and inhibited from exerting protective effects.^12^ Moreover, along with the replication of immune cell DNA, this virus is also replicated and releases more viruses to infect more cells. Finally, many types of incurable infections and tumors are generated, which lead to inevitable death owing to the lack of eligible drugs.

On the other hand, herpes zoster is related with the immune status of human body, especially with the decrease in cellular immune function. Induced by VZV [with human herpes virus 3 (HHV3) as the main pathogen], herpes zoster is clinically manifested as local nerve involvement and obvious neuralgia.^13^ During onset, this virus is activated again because of decrease in resistance or cellular immune function, which inflames involved ganglia. Notably, HIV infection remarkably increases the risk of being injected with HHV3 simultaneously.

At present, viral infection is treated by interferon in most cases mainly by fighting against virus, cell proliferation and hepatic fibrosis as well as by regulating immunity. For instance, IFN α-2b can directly inhibit the replication of hepatitis B virus.^14^ Besides improving immune function, interferon can also enhance macrophage phagocytosis, the killing effect of cytotoxic T cells and the function of natural killer cells. Nevertheless, interferon only has short-term effects while being dose-dependent, accompanied by possible adverse reactions as a result. Foscarnet sodium is a congener of phosphonoacetic acid, and they are both the analogues of pyrophosphates. It has previously been held that foscarnet sodium was an inhibitor of herpes simplex virus DNA polymerase, with its antiviral activities verified against cells *in vitro* and against guinea pigs *in vivo*.^15^ After four weeks, the effective rates of the treatment and control groups were 95.6% and 80.0% respectively, which were significantly different (P < 0.05), suggesting that foscarnet sodium effectively relieved the herpes zoster symptoms.

HIV infection complicated with herpes zoster is mainly characterized by spontaneous, needle-like, burning-like, paroxysmal pain, being different from the persistent pain caused by lumbar diseases. Hence, pain scores are usually high. When the disease occurs, patients undergo insufferable burning and itching together with neuralgia, thus being sleep-deprived in extreme cases. Approximately 14.0% of herpes zoster develops into postherpetic neuralgia as neuropathic pain that will result in severe outcomes if not treated in time. Unlike interferon that exerts only short-term effects,^16^ foscarnet sodium, which is a totally synthesized small molecule compound, functions at the pyrophosphate binding site of viral DNA polymerase. It also prevents pain sequelae by rapidly inhibiting the congestion, edema and necrosis of ganglia and corresponding sensory fibers and by preventing desmoplasia. In this study, the pain scores of the two groups were insignificantly different before treatment, but the scores of the treatment group were significantly lower than those of the control group two and four weeks after treatment (P < 0.05) as well as were significantly lower than those before treatment (P < 0.05).

Furthermore, it is well-known that CD4+ cells play an important role in maintaining immune function as the center of immune response, and HIV mainly invades human CD4+ T lymphocytes by reducing their counts and by causing functional defects. HIV infection complicated with herpes zoster can decrease CD4+ cells by hindering their maturation, and lead to indirect damages by producing autoimmune response and by inducing the apoptosis of such cells. In the meantime, Th cell-assisted or mediated immune response functions are indirectly weakened, including decrease in IL-2 production and the failure of Th cells to activate specific antigens. Acute infection is typified by plummet in the number of CD4+ T lymphocytes, which may self-recover completely or partially in most patients without special treatment. Herein, the numbers of CD4+ cells and the contents of IL-2 of both groups two and four weeks after treatment significantly exceeded those before treatment (P < 0.05), with significant inter-group differences also (P < 0.05), indicating that foscarnet sodium effectively suppressed viral infection and proliferation by regulating immune and inflammatory mechanisms. Basic and clinical studies also showed that foscarnet sodium was able to well inhibit hepatitis B virus and the activities of T, B lymphocytes.^17^ In addition, most patients tolerate foscarnet sodium well, without undergoing renal function damages if given enough water therewith.^18^ In this study, the treatment group scored significantly higher in physical activity, energy, sleep, social life and emotional reaction than the control group did two and four weeks after treatment (P < 0.05).

In short, HIV-infected patients are prone to being complicated with herpes zoster. Foscarnet sodium injection, compared with interferon, better improves the clinical outcomes and prognostic quality of life by effectively alleviating pain and by regulating immune mediated inflammatory diseases.
